# Quality of life after third molar surgery: a bibliometric analysis of randomized clinical trials

**DOI:** 10.4317/medoral.27516

**Published:** 2025-08-16

**Authors:** Eduardo Frederico Eduardo Maferano, José Lima Silva Júnior, Thyciana Rodrigues Ribeiro, Paulo Ricardo Martins-Filho, Paulo Goberlânio de Barros Silva, Ana Flávia Granville-Garcia, Fábio Wildson Gurgel Costa

**Affiliations:** 1 DDS, MSc, PhD student, Department of Dentistry, Faculty of Health Sciences, Zambeze University, Tete City, Mozambique; 2 DDS, MSc student, Postgraduate Program in Dentistry, State University of Paraíba, Campina Grande, Paraíba, Brazil; 3 DDS, MSc, PhD, Postgraduate Program in Dentistry, Faculty of Dentistry, Federal University of Ceará, Ceará, Brazil; 4 DDS, MSc, PhD. Investigative Pathology Laboratory, Federal University of Sergipe, Sergipe, Aracaju, Brazil; 5 DDS, MSc, PhD. Division of Oral Pathology, School of Dentistry, Christus University Center, Fortaleza, Ceará, Brazil; 6 DDS, MSc, PhD, Postgraduate Program in Dentistry, State University of Paraíba, Campina Grande, Paraíba, Brazil

## Abstract

**Background:**

This study aimed to conduct a bibliometric analysis of randomized controlled trials (RCTs) that evaluated quality of life following third molar surgery.

**Material and Methods:**

A bibliometric analysis was performed in accordance with the BIBLIO checklist across 11 databases: Web of Science, MEDLINE, EMBASE, CINAHL, PsycINFO, The Cochrane Library, Livivo, LILACS, Scopus, Epistemonikos, and Google Scholar. RCTs that assessed quality of life after third molar surgery were included. Searches were conducted on September 20, 2024, and updated on May 5, 2025.

**Results:**

The initial search retrieved 4,217 records, of which 46 articles were included. The average annual number of publications between 2008 and 2024 was 2.7. A total of 192 authors contributed to the included studies, with the University of Mosul being the most productive institution. India and Iraq were the most prolific countries, each contributing five publications. Most articles were published in journals specializing in oral and maxillofacial surgery. The terms extracted from the titles co-occurrence analysis revealed distinct thematic clusters.

**Conclusions:**

Scientific output on quality of life following third molar surgery has shown consistent growth. Future RCTs are encouraged to incorporate qualitative approaches to gain a broader understanding of the clinical and social impacts of surgical interventions.

** Key words:**Bibliometrics, oral health-related quality of life, third molars, randomized controlled trial.

## Introduction

In recent decades, clinical research in dentistry has undergone a paradigmatic shift: the success of an intervention is now measured not only by objective clinical parameters but also by the patient's subjective experience throughout the therapeutic process [1]. In this context, oral health-related quality of life (OHRQoL) has emerged as a central outcome, particularly in surgical procedures that involve pain, functional discomfort, and limitations in daily activities. Among these interventions, third molar removal surgery—especially mandibular extractions—has attracted increasing interest, not only due to its prevalence but also because of the significant acute impact it has on patients' daily routines and well-being during the early stages of recovery [1,2].

OHRQoL is a multidimensional construct that reflects the impact of oral conditions on an individual's daily life, encompassing physical, psychological, and social aspects, as well as perceived pain and discomfort [2,3]. Assessing this outcome, typically through validated questionnaires, provides a broader understanding of treatment outcomes, extending beyond healing and the absence of complications, and emphasizing the patient's perspective as a key element in clinical decision-making. The application of this approach in randomized clinical trials (RCTs) has enabled a more holistic evaluation of therapeutic effectiveness, particularly in contexts such as third molar extractions, where factors like trismus, edema, and pain can significantly impact the patient's life [1,3].

As interest in OHRQoL as a relevant clinical outcome grows, several RCTs have incorporated this variable as either a primary or secondary point of evaluation. However, despite the expansion of this body of literature, no studies have yet synthesized the profile of scientific production focused on OHRQoL following third molar extractions through bibliometric analysis. Bibliometric analysis, by quantifying patterns of publication, collaboration, and impact, serves as a robust tool for understanding the development of a research field and identifying its gaps and emerging trends [4].

In this context, the present study aims to conduct a comprehensive bibliometric analysis of RCTs evaluating quality of life after third molar surgery, focusing on the temporal evolution of scientific production, the most productive authors, institutions, and countries, the most influential journals, the most frequent keywords in titles, and the most cited articles. By mapping this landscape, the study seeks to provide a systematized and up-to-date view of the importance placed on OHRQoL in the scientific literature, offering valuable insights for future research and patient-centered clinical practices.

## Material and Methods

- Study design

This study employed a bibliometric approach, following the checklist of the Guidelines for Reporting Bibliometric Reviews of Biomedical Literature (BIBLIO) [4], to analyze the characteristics of the literature on quality of life assessment after third molar extraction, with a specific focus on RCTs.

- Data collection

Data for this study were collected from eleven databases, namely: Web of Science (Clarivate Analytics), MEDLINE (via PubMed), EMBASE, CINAHL, PsycINFO, The Cochrane Library, Livivo, LILACS, Scopus, Epistemonikos, and Google Scholar (for grey literature) on April 29, 2025, with a subsequent update of the search conducted on May 12, 2025.

- Search strategy

The search strategy employed in this study included a comprehensive selection of keywords and MeSH terms, such as "third molars" and "quality of life," along with their synonyms. These terms were combined using Boolean operators: "AND" to link primary concepts and "OR" to combine synonyms. The strategy was pre-tested and refined through discussions. It was further tailored to the specific indexing terms and search syntax of each database (see Supplement 1 for full details). Filters were applied to retrieve only articles, without restrictions on the publication year or language, ensuring an exhaustive collection of all relevant evidence.

- Eligibility criteria

- This bibliometric review included RCTs with parallel, crossover, or split-mouth designs that assessed quality of life as an outcome after third molar surgery, regardless of the statistical significance of the primary outcomes. RCTs that evaluated quality of life at a single time point or over multiple follow-up periods were considered eligible. Studies were excluded if they involved third molar surgery in pregnant or lactating women, patients with pre-existing cognitive impairment, or if they were published as brief communications or research letters.

- Screening

After retrieving records from the eleven databases, all references were exported to EndNote Web (Clarivate Analytics, PA, USA) for initial duplicate removal. Subsequently, the records were imported into the Rayyan platform (http://rayyan.qrci.org, Qatar Foundation, Qatar), where a second round of duplicate screening was performed to enhance accuracy.

After deduplication, two independent reviewers (EFEM and AFGG) screened the titles and abstracts according to predefined eligibility criteria to identify potentially relevant articles for full-text evaluation. In the second phase, full-text articles were assessed based on the same criteria, and reasons for exclusion were systematically recorded and presented in a PRISMA flow diagram.

To minimize bias during the study selection process, interrater agreement was calculated using the Kappa statistic, which indicated an excellent level of concordance (Kappa = 0.97). Any discrepancies were resolved by consensus with a third reviewer (FWGC), a methodologist experienced in scope reviews and an expert in the field.

- Data extraction

Data extraction was performed by two independent, pre-trained reviewers (EFEM and AFGG) for all studies included in this bibliometric analysis. The following information was collected for each article: authors' names, country and continent of origin, year of publication, journals where the articles were published, article title and keywords, institutions involved in the study, and the number of citations acquired from Google Scholar for each article.

- Data analysis

The data were described using absolute and relative frequencies. A Poisson regression model with a logarithmic link was adjusted to assess the temporal trend of occurrences (α = 0.05). Word cloud graphics and co-occurrence maps were generated based on the words in the titles. The authors' h-index was retrieved from Scopus, and the number of citations for the articles was obtained from Google Scholar. The database was manipulated in Microsoft Excel, and the analyses were performed using R software, version 4.3.2, with the Bibliometrix package.

## Results

- Search strategy and study selection

**Figure 1:**
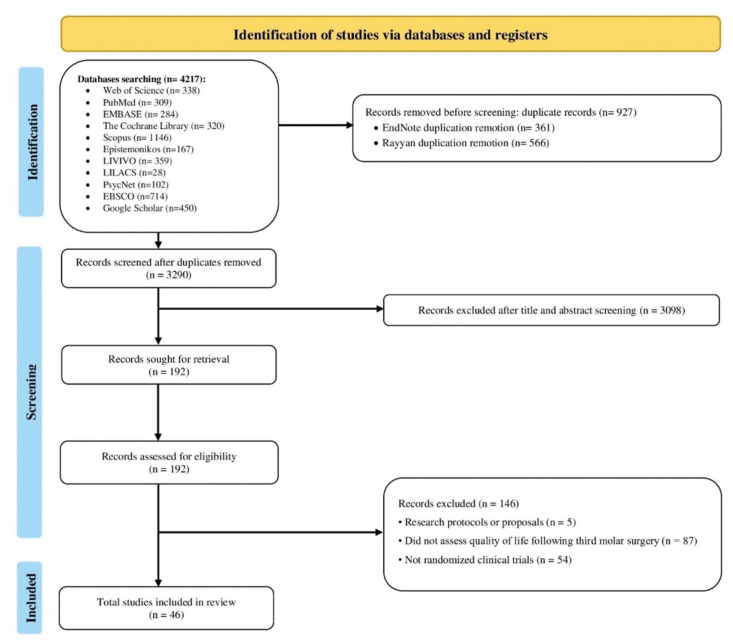
Flow diagram of literature search and selection criteria.

The search strategy identified a total of 3,767 records across ten electronic databases, in addition to 450 additional records retrieved from grey literature sources, specifically Google Scholar. After removing 927 duplicates using EndNote and Rayyan software, 3,290 unique records remained for title and abstract screening. Based on the predefined eligibility criteria, 192 articles were selected for full-text reading. After obtaining the full texts and a thorough assessment according to the eligibility criteria, 146 articles were excluded: five were research protocols, 87 did not assess quality of life after third molar surgery, and 54 were not RCTs. Ultimately, 46 studies met the inclusion criteria and were incorporated into the final analysis, as illustrated in Fig. 1.

- Bibliometric analysis

- Trends in Annual Publications

**Figure 2:**
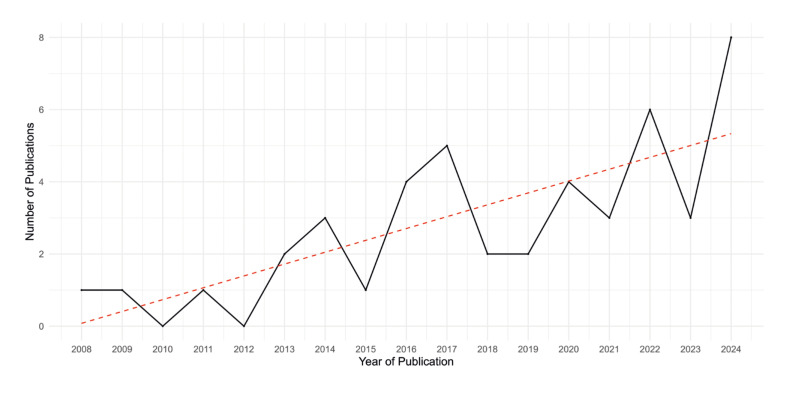
Trends in annual publication.

Table 1 presents the total number of publications and the annual percentage variation between 2008 and 2024. The average annual number of publications during this period was 2.7, with a standard deviation of 2.2. Poisson regression analysis revealed a positive and significant association between the year and the number of publications (Exp(β) = 1.11; 95% CI: 1.04 - 1.20; *p* = 0.002), indicating an average annual increase of 11.3% in the number of publications. The time series plot is presented in Fig. 2.

- Author profile

**Figure 3:**
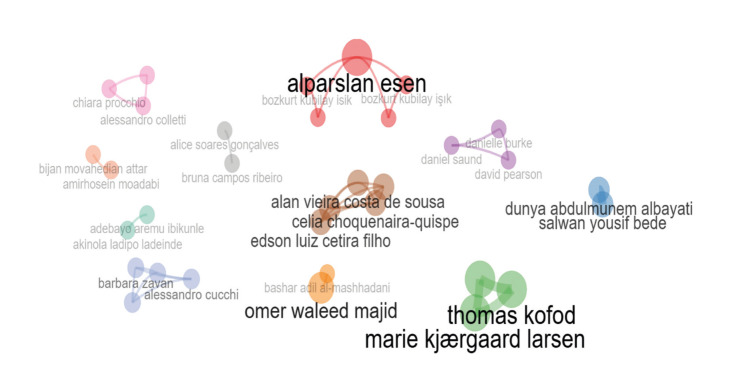
The collaboration network between the authors.

A total of 192 authors were identified in the analyzed publications. The author Omer Waleed Majid (Iraq) was the most frequent, with three publications, accounting for 6.5% of the total. Other authors, such as Salwan Yousif Bede (Iraq), Thomas Kofod (Denmark), Thomas Starch-Jensen (Denmark), Dunya Abdulmunem Albayati (Iraq), and Marie Kjærgaard Larsen (Denmark), contributed two articles each, representing 4.3% of the total. The remaining authors participated in only one article each. More details can be found in Table 2. Fig. 3 illustrates the collaboration network between the authors.

- Institution distribution

Among the participating institutions, the University of Mosul was the most prolific, contributing three studies (6.5%). Five institutions followed with two publications each (4.3%): Aalborg University Hospital, Damascus University, Southern Medical University, Necmettin Erbakan University, and University of Baghdad. These data reveal a relatively homogeneous distribution among the most productive institutions, without the prominent dominance of a single institution in Table 2.

- Geographical distribution of scientific output

**Figure 4:**
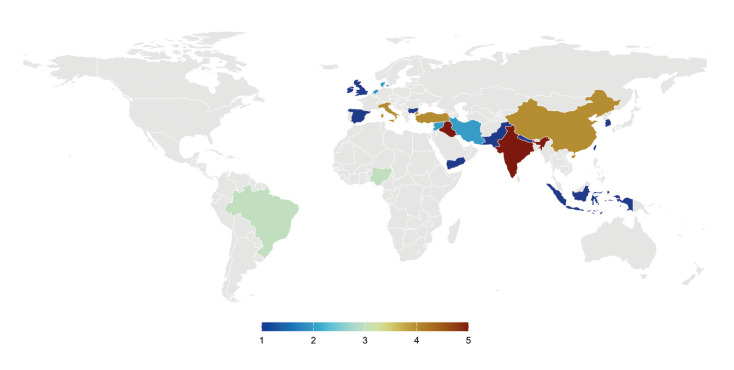
Geographical distribution of scientific output.

Regarding geographical distribution, India and Iraq stood out as the most productive countries, with five publications each (10.9%). Following them, China, Italy, and Turkey contributed four studies each (8.7%), demonstrating a significant contribution to the analyzed literature. Brazil and Nigeria contributed three publications each (6.5%), while Denmark, the Netherlands, Iran, and Syria recorded two publications (4.3%) each. Other countries, such as Bulgaria, South Korea, Spain, Yemen, Indonesia, Ireland, Nepal, Pakistan, the United Kingdom, and Taiwan, had only one publication (2.2%), indicating a more limited participation in the field under investigation. Further details on these data are available in Table 3 and Fig. 4.

- Article citation analysis

The analysis of the most cited publications revealed that the study by Kazancioglu *et al*. (2014), titled "Effects of ozone therapy on pain, swelling, and trismus following third molar surgery," is the most cited, with 154 references. In second place, Majid’s (2011) study, which addressed the effect of submucosal dexamethasone on quality of life after third molar surgery, received 142 citations. Majid also appears in third and tenth positions, with significant contributions to the field, being cited 114 times in the study on the perioperative use of bromelain (Majid & Al-Mashhadani, 2014) and 49 times in the research comparing different routes of dexamethasone administration (Majid & Mahmood, 2013). Another relevant study is by Warraich *et al*. (2013), with 113 citations, which evaluated the use of submucosal dexamethasone in a randomized, double-blind, prospective study. Fifth, the work by Piersanti *et al*. (2014), comparing piezosurgery with conventional rotary instruments, received 102 citations. Other noTable studies include research on cryotherapy (Forouzanfar *et al*. 2008), corticosteroid therapy (Chugh *et al*. 2017), antibiotics (Limeres *et al*. 2009), and oral bromelain (Ghensi *et al*. 2017), with citations ranging from 55 to 94. More details are presented in Table 4.

- Journal distribution

It was observed that most publications were concentrated in journals specialized in oral and maxillofacial surgery. The Journal of Oral and Maxillofacial Surgery was the most frequent journal, with 17.4% (n = 8) of the publications, followed by Oral and Maxillofacial Surgery, with 13.0% (n = 6). Clinical Oral Investigations, International Journal of Oral & Maxillofacial Surgery, and Medicina Oral Patología Oral y Cirugía Bucal each had a frequency of 8.7% (n = 4).

The remaining publications (n = 20; 43.5%) were distributed across various journals with lower individual frequency and varied or unreported impact factors. These data suggest that the scientific output is predominantly concentrated in journals with an impact factor between 1.7 and 3.1, highlighting the relevance of the investigated topic and the preference for publishing in clinically and methodologically qualified journals.

- Term frequency analysis based on word cloud representation

The word cloud illustrates the frequency of terms extracted from the titles of the analyzed studies, with larger font sizes indicating higher frequency. The most central terms reflect the most recurrent themes in research related to the assessment of quality of life after third molar surgery. This analysis is presented in Supplement 2.

-Word co-occurrence network

The analysis of the word co-occurrence network extracted from the research titles revealed distinct thematic clusters. Nodes represent the most frequent words, while connections indicate their co-occurrence in the same titles. The size of the nodes reflects the centrality of each term, while colors group related words. Additional details are provided in Supplement 3.

Cluster 1 (Red): Dominated by the term "trial," with high centrality, indicating that this group of studies focuses on RCTs. Other terms, such as "mandibular," "impacted," "controlled," and "molars," suggest that the studies primarily address the extraction of impacted lower third molars and the evaluation of effects such as pain and trismus.

Cluster 2 (Blue): The largest and most centralized, highlighting the terms "molar," "surgery," and "randomized." This group also includes terms related to quality of life ("life," "quality," "health-related"), postoperative effects ("postoperative," "discomfort," "complications"), and drug administration methods ("oral," "submucosal," "injection," "dexamethasone"), reflecting a focus on clinical studies on third molar surgery and adjunctive pharmacological interventions.

Cluster 3 (Green): A smaller but cohesive group, gathering terms like "flap," "conventional," "envelope," and "piezosurgery," indicating interest in specific surgical techniques, possibly comparing conventional approaches with methods such as piezoelectric surgery.

Further relevant information related to the present analysis is available in Supplement 4, Supplement 5, and Supplement 6.

## Discussion

Bibliometric analysis is a well-established tool for assessing the impact of scientific output through indicators such as the Journal Impact Factor (JIF) and citation count. This approach enables the identification of publications that influence clinical practice, inform public health policies, and guide future research directions [5]. In dentistry, bibliometric methods have been applied to a wide range of topics, including facial trauma, oral cancer, orthognathic surgery, and preemptive analgesia [6-9]. To the best of our knowledge, this is the first study to apply the BIBLIO guidelines [4] in a bibliometric analysis focused exclusively on RCTs evaluating quality of life outcomes following third molar surgery. This approach enabled the mapping of scientific production trends, identification of the most prolific authors and institutions, and highlighting of the most influential articles, keywords, and titles in the field.

- Publication trends

The analysis revealed an average annual growth rate of 11.3% in the number of publications, indicating increasing interest in the topic. This trend aligns with previous findings from reviews on health-related quality of life [10], reflecting the growing emphasis on patient-centered outcomes as markers of therapeutic success. These results underscore the central role of OHRQoL in both clinical practice and RCTs within oral and maxillofacial surgery.

- Most productive authors, institutions, and countries

Iraq and Denmark emerged as the most productive countries, contrasting with the traditionally dominant roles of the United States, United Kingdom, Germany, and Canada in global quality of life research [10]. This deviation may reflect these countries’ specific engagement in surgical OHRQoL research. The University of Mosul was identified as the most productive institution, indicating active scientific networks focused on clinical outcomes and postoperative well-being. Compared to Zheng *et al*. [10], the present findings suggest a greater incidence of international collaborations, reinforcing the multidisciplinary and globalized nature of this research area.

- Most influential journals

Most publications appeared in specialized journals such as the Journal of Oral and Maxillofacial Surgery and Oral and Maxillofacial Surgery. The preference for high-impact journals suggests a strategic effort by authors to increase visibility and prestige within the academic community. Similar trends have been observed in other areas of dentistry, such as dental materials [11], demonstrating a link between thematic specialization and strategic journal selection to enhance academic impact.

- Frequency of terms extracted from the titles

The clustering of the most frequent terms extracted from the titles revealed a thematic concentration around terms such as “dexamethasone,” “pain,” “trismus,” and “quality of life.” The recurrence of terms like “molar,” “surgery,” and “randomized” further underscores the focus on RCTs examining surgical interventions and their effects on patient well-being. The prominence of pharmacological adjuncts, such as dexamethasone, reflects a trend toward optimizing postoperative management. These findings indicate a maturing scientific literature increasingly oriented toward clinically relevant outcomes. The systematic review by Almeida *et al*. [12] supports this trend, highlighting the efficacy of corticosteroids in reducing pain, swelling, and trismus, thereby validating their use in enhancing postoperative recovery.

- Most cited articles

Citation count remains a key indicator of scientific recognition. In this analysis, only five articles exceeded 100 citations—a threshold often used to classify a publication as “classic” [13]. The most cited studies, such as those by Kazancioglu *et al*. [14] and Majid [15], explored pharmacological strategies including dexamethasone and bromelain aimed at improving patient comfort and recovery. While citation counts reflect visibility and relevance, they do not necessarily indicate methodological rigor [16]. Nonetheless, the present findings underscore the growing importance of quality of life as a clinical outcome in oral surgery. Strategies such as dexamethasone use, supported by the literature, stand out as effective options for postoperative symptom control. There remains a need for well-designed RCTs investigating novel interventions using validated instruments such as the OHIP-14 and PoSSe [1,17], ensuring accurate measurement of patient-reported experiences.

Despite the methodological breadth of this study—including a search across eleven scientific databases and strict adherence to the BIBLIO guidelines—certain limitations should be acknowledged. Bibliometric analyses depend on the accuracy and consistency of indexed data and may be affected by biases such as lower citation counts for more recent publications that have had limited time to accumulate references. Furthermore, the exclusive use of quantitative metrics may overlook qualitative aspects of research, including methodological validity, clinical applicability, or scientific innovation.

Although the exclusive inclusion of RCTs ensures the analysis of high-level evidence, this criterion may have limited the scope of our findings by excluding relevant data from observational studies and other research designs.

A further limitation of this bibliometric analysis is that it did not include a critical appraisal of the methodological quality or risk of bias of the included RCTs. While such assessments are essential in systematic reviews and meta-analyses, they are not standard components of bibliometric studies. Nonetheless, the lack of internal validity appraisal should be taken into account when interpreting the influence and quality of the included studies.

We also observed a predominance of research output from specific countries, which may reflect regional research priorities, publication incentives, and increased academic engagement with the topic. While this distribution provides valuable insight into global interest in postoperative quality of life, it may also limit the generalizability of the findings to other healthcare settings. These patterns are likely influenced by differences in research infrastructure, funding availability, and access to indexed journals across regions.

## Conclusions

This bibliometric analysis demonstrated a consistent growth in scientific output concerning quality of life following third molar surgery, with emphasis on patient-centered therapeutic strategies, consolidated thematic patterns, and widespread international collaboration. The findings confirm the relevance of OHRQoL as a critical clinical outcome in oral and maxillofacial surgery.

Based on these results, it is recommended that future RCTs prioritize the use of validated instruments such as the OHIP-14 and PoSSe, expand the diversity of clinical contexts assessed, and incorporate qualitative approaches to better capture the clinical and societal impact of interventions. Additionally, future bibliometric studies may benefit from including a broader range of study types to provide a more comprehensive view of the literature. Such initiatives will contribute to strengthening the scientific foundation and advancing patient-centered care in oral surgery.

## Figures and Tables

**Table 1:** Number of publications and annual percentage growth (2008-2024).

Year of publication	n	%	Annual percentage variation
2008	1	2.2%	-
2009	1	2.2%	0.0%
2010	0	0.0%	-100.0%
2011	1	2.2%	-
2012	0	0.0%	-100.0%
2013	2	4.3%	-
2014	3	6.5%	50.0%
2015	1	2.2%	-66.7%
2016	4	8.7%	300.0%
2017	5	10.9%	25.0%
2018	2	4.3%	-60.0%
2019	2	4.3%	0.0%
2020	4	8.7%	100.0%
2021	3	6.5%	-25.0%
2022	6	13.0%	100.0%
2023	3	6.5%	-50.0%
2024	8	17,4%	166.7%

**Table 2:** Distribution of leading authors.

Leading authors	n	%	Institution	Country	H-index
Omer Waleed Majid	3	6.5%	University of Mosul	Iraq	10
Salwan Yousif Bede	2	4.3%	Aalborg University Hospital	Iraq	8
Thomas Kofod	2	4.3%	Damascus University	Denmark	10
Thomas Starch-Jensen	2	4.3%	Southern Medical	Denmark	14
Dunya Abdulmunem Albayati	2	4.3%	Necmettin Erbakan	Iraq	-
Marie Kjærgaard Larsen	2	4.3%	University of Baghdad	Denmark	3

**Table 3:** Most productive countries in publications.

Most productive countries	n	%
India	5	10.9%
Iraq	5	10.9%
China	4	8.7%
Italy	4	8.7%
Turkey	4	8.7%
Brazil	3	6.5%
Nigeria	3	6.5%
Denmark	2	4.3%
Netherlands	2	4.3%
Iran	2	4.3%
Syria	2	4.3%
Bulgaria	1	2.2%
South Korea	1	2.2%
Spain	1	2.2%
Yemen	1	2.2%
Indonesia	1	2.2%
Ireland	1	2.2%
Nepal	1	2.2%
Pakistan	1	2.2%
United Kingdom	1	2.2%
Taiwan	1	2.2%

**Table 4:** Articles with the highest number of citations.

Author	Article title	Citations
Kazancioglu et al. 2014	Effects of ozone therapy on pain, swelling, and trismus following third molar surgery	154
Majid, 2011	Submucosal Dexamethasone Injection Improves Quality of Life Measures After Third Molar Surgery: A Comparative Study	142
Majid & Al-Mashhadani, 2014	Perioperative Bromelain Reduces Pain and Swelling and Improves Quality of Life Measures After Mandibular Third Molar Surgery: A Randomized, Double-Blind, Placebo-Controlled Clinical Trial	114
Warraich et al. 2013	Evaluation of postoperative discomfort following third molar surgery using submucosal dexamethasone - a randomized observer blind prospective study	113
Piersanti et al. 2014	Piezosurgery or Conventional Rotatory Instruments for Inferior Third Molar Extractions?	102
Forouzanfar et al. 2008	Effect of ice compression on pain after mandibular third molar surgery: a single-blind, randomized controlled trial	94
Chugh et al. 2017	Submucosal injection of dexamethasone and methylprednisolone for the control of postoperative sequelae after third molar surgery: randomized controlled trial	69
Limeres et al. 2009	Patients' Perception of Recovery After Third Molar Surgery Following Postoperative Treatment With Moxifloxacin Versus Amoxicillin and Clavulanic Acid: A Randomized, Double-Blind, Controlled Study	56
Ghensi et al. 2017	Effect of Oral Administration of Bromelain on Postoperative Discomfort After Third Molar Surgery	55
Majid & Mahmood, 2013	Use of dexamethasone to minimise post-operative sequelae after third molar surgery: comparison of five different routes of administration	49
